# A non-parametric significance test to compare corpora

**DOI:** 10.1371/journal.pone.0222703

**Published:** 2019-09-19

**Authors:** Alexander Koplenig

**Affiliations:** Leibniz Institute for the German language (IDS), Mannheim, Germany; University of Aveiro, NEW ZEALAND

## Abstract

Classical null hypothesis significance tests are not appropriate in corpus linguistics, because the *randomness* assumption underlying these testing procedures is not fulfilled. Nevertheless, there are numerous scenarios where it would be beneficial to have some kind of test in order to judge the relevance of a result (e.g. a difference between two corpora) by answering the question whether the attribute of interest is pronounced enough to warrant the conclusion that it is substantial and not due to chance. In this paper, I outline such a test.

## 1 Introduction

“Their function is to prevent us being deceived by accidental occurrences, due not to causes we wish to study, or are trying to detect, but to a combination of the many other circumstances which we cannot control.”

Ronald Aylmer Fisher, 1929 [1]

In a recent comment in Nature that was published along with a letter signed by more than 800 researchers, the co-signatories call to retire ‘statistical significance’[2]. It is part of an ongoing lively debate among statisticians and quantitative researchers [3–10] to find ways to ‘fix statistics’ [11] (partly) in response to the current reproducibility crisis [12–16] in psychology [17,18], experimental economics [19] and social science [20]. In this context, the prevalent so called null hypothesis significance testing paradigm has increasingly come under attack due to widespread misunderstandings, misapplications and misconceptions, as [21] puts it: “*P* values linked to null hypothesis significance testing (NHST) is the most widely (mis)used method of statistical inference.”

In corpus linguistics, a discipline that studies human language based on corpora, i.e. large collections of samples of “real life” language use (e.g. textual data or transcripts of spoken data) [22], the use of null hypothesis significance testing is also a very common standard approach. In a recent paper, I argued that corpus linguists should abandon null hypothesis statistical significance testing [23]. However, I did not reach this conclusion, because of the problems with the testing paradigm discussed in the papers cited above or because of the special requirements of corpus linguistic data [24–28], but, following [29], because the assumptions on which these tests are based are not fulfilled in corpus linguistics for reasons of principle.

To summarize my argument again, in commonly used tests of statistical significance the *p-value* is the probability of observing a result that is equal, or more extreme, than the one we found in sample of size *n*, given the fact that there is actually no relationship in the population, i.e. the null hypothesis is true. An inferential statistical test is always a statement about a population not about a sample [30; p. 392]. A crucial, but often overlooked assumption underlying these testing procedures is *randomness* [31–38], which can either be achieved by (i) randomly assigning subjects to different experimental conditions or by (ii) randomly sampling elements from the population [35]. Without randomness, null hypothesis significance testing becomes meaningless, because we cannot use probability theory to address the sampling error, i.e. the degree of error due to chance [33]. Or put differently, “random samples” are one of the “building blocks for hypothesis testing” [36; p. 202]. On the one hand, randomly assigning the units of interest to the experimental conditions eliminates the selection bias, which is the potential influence of confounding variables on an outcome of interest. Balancing the "subject’s characteristics across the different treatment groups" [39] ensures that the experimental condition and all possible (even unidentified) variables that could affect the outcome are uncorrelated. Through the manipulation of the independent variables, it can be inferred that, random fluctuations aside, the treatment is the cause of the outcome [40]. On the other hand, random sampling ensures that we can statistically estimate how representative a sample is of a population that is “specified in the null hypothesis” [35].

Using appropriate techniques, null hypothesis significance testing can be used if it is the goal of a corpus linguistic study to make generalizations about a large corpus based on a smaller random sample drawn from it [41]. However in many (if not most) situations, corpus linguists are not interested in the specific samples included in a corpus, but instead want to find generalizations about the studied language and its structure [22,42–47]. Correspondingly, statistical inference is used to estimate unknown population quantities based on the characteristics of a sample from it:

“It is rarely the case that linguists are interested in the samples per se, rather than in generalizations from the samples to the infinite amount of text corresponding to the extensional definition of a (sub)language. For example, a linguist studying a pattern in the 500 text samples of the Brown corpus will typically be interested in drawing conclusions about (written) American English as a whole, and not just about the specific texts that compose the Brown. Statistical inference allows the linguist to generalize from properties observed in a specific sample (corpus) to the same properties in the language as a whole” [48; p. 2]

However, the problem in this context is that *randomness* assumption is not fulfilled. Regarding (i), corpus studies are observational per definition, thus, real experimental research is not possible. Regarding (ii), I have argued in [23] that it is both not possible to define a corpus as a random sample of the language it seeks to represent [22,49] and that there is widespread consensus that corpus linguistics requires a different “notion of representativeness” [50]. Here, representativeness is typically achieved by balancing, i.e. by including a wide variety of different text types in the corpus that are “intuitively considered relevant” [51; p. 47, my translation]. However, there is “no objective way to balance a corpus” [22; p. 21], because (a) we do not know the distribution of the parameters in the population, e.g. the “true” proportion of written vs. spoken language in the corresponding “language as a whole” and (b) different researchers might have different opinions on defining different registers and sub-registers as well as genres and subgenres that should constitute the corpus. Let me emphasize that I do not claim that the balancing-approach is not a good idea, on the contrary, balanced corpora such as the Brown corpus [52], the BNC corpus [53] or the COHA corpus [54] constitute an invaluable source of information not only for corpus and computational linguistics, but for linguistics in general. I am merely claiming that for linguistic corpora (balanced or not) the *randomness* assumption is not fulfilled, because the elements of study (words, phrases, sentences, etc.) are neither *randomly assigned* to different experimental conditions nor are they *randomly drawn* from the population they seek to represent (i.e. a language or language variety).

However, this has important ramifications for the quantitative analysis of corpus data, because, as written above, null hypothesis significance tests, are based on exactly this assumption:

“Conventional statistical inferences (e.g., formulas for the standard error of the mean, *t* -tests, etc.) depend on the assumption of random sampling. This is not a matter of debate or opinion; it is a matter of mathematical necessity.” [37; p. 2]

“It is not possible to estimate the error with accidental sampling and many other sampling strategies since they contain unknown types and degrees of bias in addition to sampling error.” [36; p. 177]

When discussing my paper and the arguments contained therein with my colleagues on several occasions, one question came up every time: What do you recommend if we want to judge the relevance of a result that is based on corpus linguistic data? Or, in relation to the introductory quote, what can we do to separate the wheat from the chaff, where the former relates to the effects of the ‘causes we wish to study’ and the latter to the ‘accidental occurrences’ that are the ‘result of a combination of the many other circumstances which we cannot control’?

Giving a potential answer to this question is the main objective of this paper. To this end, the remainder of this paper is structured as follows: The data is presented in the next section (Section 2). I will then present several typical corpus linguistic examples where the need for some kind of ‘separation test’ is evident, but standard parametric tests do not work (Section 3). I will demonstrate that *permutation tests* can be used in situations where we do not want to use statistical inference to estimate unknown population quantities based on sample drawn from it, but want to judge the relevance of an outcome based on corpus linguistic data in Section 4. The results are presented in Section 5. In Section 6, I will show that, under certain circumstances, it is possible to use standard testing procedures to approximate the results of a *permutation test*. The paper ends with some concluding remarks in Section 7.

## 2 Data

In this paper, I use the complete works of William Shakespeare and the complete works of Jane Austen. The Unicode versions of both works were taken from the Project Gutenberg [55,56]. Boilerplates were removed manually. To lemmatize the texts, I used the TreeTagger with an English parameter file [57]. Punctuation and cardinal numbers were removed and all characters were converted to lowercase. Each token of a word is replaced by its corresponding lemma. For illustrative reasons, I will continue to use the term ‘word’ instead of ‘lemma’ in what follows. In total, the corpus size, denoted as *C*, of the Shakespeare corpus is 913,730 words. The size of the Austen corpus is 779,331 words.

Stata (14) code required to reproduce all results presented in this paper is available as electronic supplementary material (S1 Code).

## 3 Quantifying differences

Imagine the aim of a study was to compare the lexical richness/diversity between Shakespeare and Austen. One standard way to estimate the lexical richness for a corpus or a text is to calculate the type-token ratio that is defined as the number of different word types divided by the overall number of word tokens [58]:

$${TTR}_{i}=\frac{V(C)}{C}$$ (1)

where *V(C)* denotes the number of types in a corpus *i* of *C* tokens. However, this measure is known to be strongly affected by the corpus size as demonstrated by [59]. One way to solve this problem was first described by [60]. He suggests first dividing the corpus into *I* = ⌊*C*/*N*⌋ segments of *N* words (*NB*.: Since the modulus of *C* with respect to *N* will in most cases be greater than zero, the remainder, i.e. the last segment starting at *C*-*N*+1 and ending at *C* is usually dropped). For each segment, *TTR* is calculated. Then the *mean segmental TTR* is computed by averaging the values of each segment:

$$\begin{array}{c} M_{i}=\frac{N}{C}*\left[\frac{{V(N)}_{1}^{N}}{N}+\frac{{V(N)}_{N+1}^{2N}}{N}+\frac{{V(N)}_{2N+1}^{3N}}{N}+\cdots +\frac{{V(N)}_{(I-1)N+1}^{IN}}{N}\right]\\ =\frac{1}{C}\sum_{i=1}^{I}{V(N)}_{(i-1)N+1}^{iN} \end{array}$$ (2)

where ${V(N)}_{x}^{y}$ denotes the number of types that occur in the substring of the corpus starting at the *x*th word and ending at the *y*th word. Because of the associated intuitiveness, one obvious choice of *N* would be to choose a number that approximates the average word count per page of a book. With a 12-point font and double spacing, *N* could be 250 words. With this standard words-per-page count, we have 3,117 pages for the Austen ‘book’ and 3,654 pages for the Shakespeare ‘book’. For the comparison of the *TTR* values, *M* = 0.563 for Austen and *M* = 0.567 for Shakespeare.

This illustrates a situation where the researcher might ask if the rather small difference of 0.004 is *accidental* or if it is *substantial* enough to warrant the conclusion that the lexical richness of Shakespeare is larger than that of Austen.

**Fig 1:**
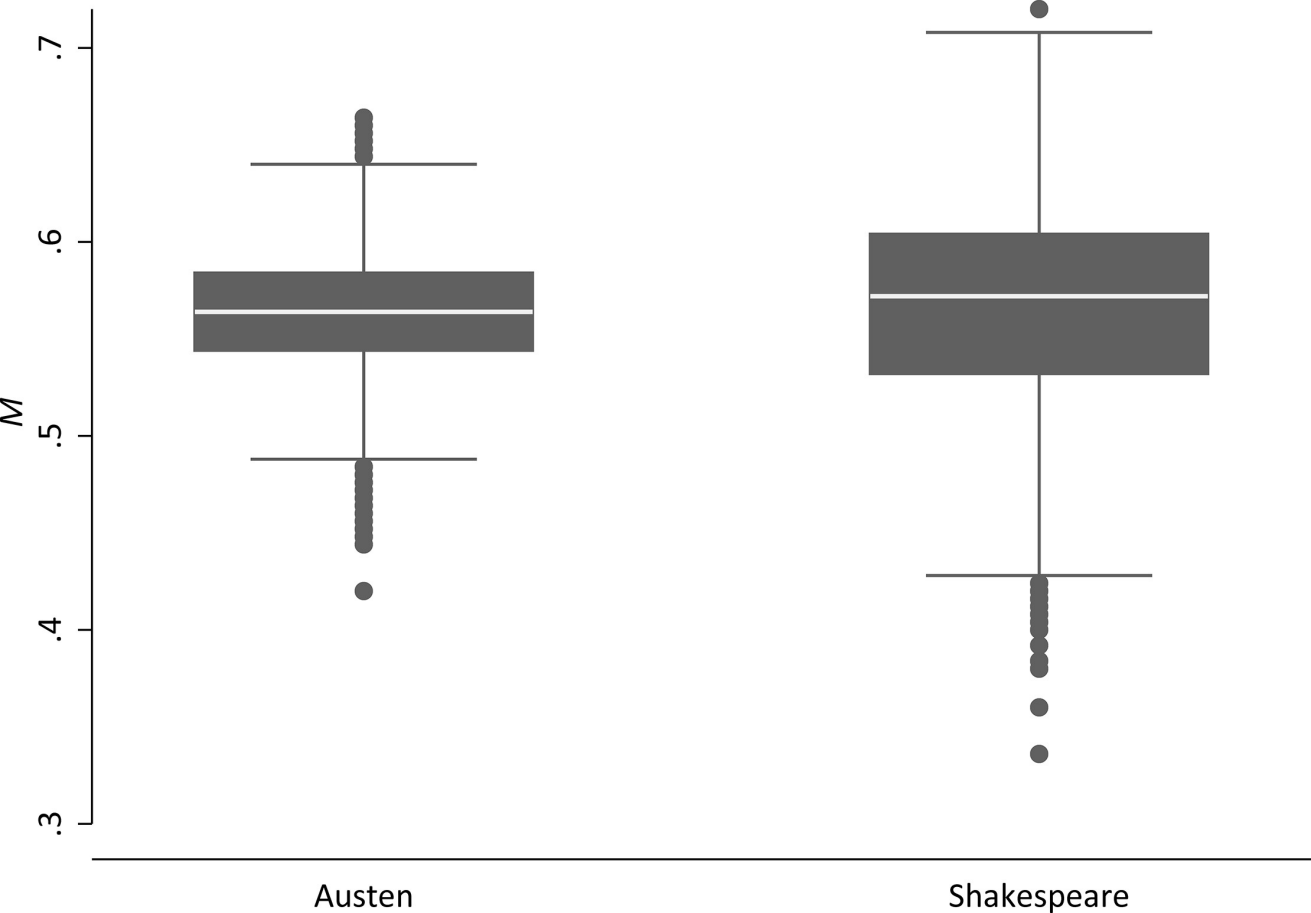
*TTR* values per page for the Austen corpus (3,117 *pages*) and the Shakespeare corpus (3,654 *pages*). *NB*.: A page is defined as consisting of *N* = 250 words.

It is interesting to note that by simply aggregating the *TTR* values of each page as suggested by [60], we do not efficiently exploit the available information: Fig 1 depicts two separate boxplots for the 3,117 pages of the Austen corpus and the 3,654 pages of the Shakespeare corpus. So instead of just looking at the means, the researcher could decide to conduct a standard parametric one-sided two-sample *t*-test on the equality of means, with the *TTR* value for each page as the variable of interest and the author as the grouping variable in order to assess the difference between the two authors. Regarding the following sections, it makes sense to define the statistic of interest in relation to the standard Pearson correlation coefficient *r*:

$$t=r_{yz}\sqrt{\frac{L-2}{1-r^{2}}}$$ (3)

where *L* denotes the number of available pages in the concatenation of two corpora, here *L* = 6,771 pages. If *z* is the dichotomous grouping variable coded as 1 for the first group and 0 for the second group, then the correlation coefficient can be written as [61]:

$$r_{yz}=\frac{M_{1}-M_{0}}{s_{y}}\sqrt{\frac{L_{1}L_{0}}{L}}$$ (4)

where *s*_*y*_ denotes the standard deviation of the *y*-variable. *M*_*1*_ and *L*_1_ represent the arithmetic mean and number of cases (here: *pages*) for group 1. Likewise for group 0. In the example, *t* = 4.023. The result is distributed as Student’s *t* with *L—*2 *=* 6,769 degrees of freedom, the corresponding *p*-value shows that this difference is highly significant with a significance level that is lower than .01.

As a further illustration, I simulated a second dataset that has the same *page* distribution as the original corpora (3,117 vs. 3,654 *pages*). For Austen*, we receive a value of *M* = 0.5643 and *M* = 0.5655 for Shakespeare*. Again, the researcher might ask whether the difference of 0.0012 for this mock dataset is *accidental* or *substantial*. In this case, the *t*-test returns *p* = 0.1329. Thus, in the first case, the researcher could argue that the difference is *substantial*, while in the latter, it is only *accidental*.

However, as outlined in [23], this classical statistical significance test measures the probability of observing the data given the null hypothesis is true, i.e., *p*(Data | H_0_), where the *null hypothesis* states that there is *exactly* no relationship between the measured quantities in the population, while its ‘rival’, the *alternative hypothesis* assumes that there is a relationship. Or put differently, the *p*-value represents the probability of drawing a random sample that suggests the existence of a relationship from a population where this relationship does not exist. While, as mentioned above and in [23], corpora cannot be considered random samples, in this context the following is even more important: what is the population that the corpora are assumed to be samples of? As mentioned above, we actually analyze the complete known (written and published) works of the two authors. Thus, an analysis of those two works amounts to an exhaustive data collection process and in this context a classical test for statistical significance does not make any sense since there is no inference. We could somehow claim that the corpora represent an imaginary population of the (written) language of Austen and Shakespeare. However, as [37 fn. 2] put it:

“Such a population has no empirical existence, but is defined in an essentially circular way—as that population from which the sample may be assumed to be randomly drawn. At the risk of the obvious, inferences to imaginary populations are also imaginary.”

One anonymous reviewer suggested to treat the texts that were written by an author as a sample of the population of potential texts that the author could have written. In this case, the collected works of both Austen and Shakespeare are treated as a sample of their potential output. However, to see that the *randomness* assumption is also violated in this case, note that a standard parametric significance test answers the question what is likely to happen were the study to be repeated, without actually having to repeat it. However, if we would actually repeat the study, then again we would only sample those very texts the corresponding author has actually written which sets them apart from all texts that an author could have written, but actually did not write. Thus since all texts an author has actually written share a common feature, that of “being actually written” and because “unwritten” texts have zero chance to be drawn, the sample is biased and the randomness assumption of standard parametric tests is violated.

Even if we would find a way to make this work, it is important to point out that the purpose of the test is not to make any claims about the imaginary population based on the samples, but to:

“determine whether some attribute of data was too pronounced to be easily explained away as some kind of fluke” [62]

Consider a second example that represents a very common situation in corpus linguistics [63]: the comparsion of word frequencies. The relative frequency of a word *w* in a corpus that is *C* words long is given as:

$$p_{w}=\frac{f(w)}{C}$$ (5)

where *f*(w) denotes the absolute frequency of *w*. Let us assume that the researcher is interested in comparing the relative frequencies of the following four words between Austen and Shakespeare: *brother*, *he*, *she* and *woman*.

As in the last example, the researcher might want to determine whether any difference between those words is large enough to warrant a substantial explanation. Again, comparing just the two relative frequencies does not efficiently use the information. In analogy to the idea of [60], note that the relative frequency of a word *w* can also be written as

$$p_{w}=\frac{{f(w)}_{1}^{N}+{f(w)}_{N+1}^{2N}+{f(w)}_{2N+1}^{3N}+\cdots +{f(w)}_{C-N+1}^{C}}{C}$$ (6)

where ${f(w)}_{x}^{y}$ denotes the token frequency of *w* in the substring of a corpus starting at the *x*th word and ending at the *y*th word. Again, we divide each corpus into *I* segments of *N* words and drop the last (incomplete) segment:

$$p_{w}=\frac{1}{C}\sum_{i=1}^{I}{f(w)}_{(i-1)N+1}^{iN}$$ (7)

**Fig 2:**
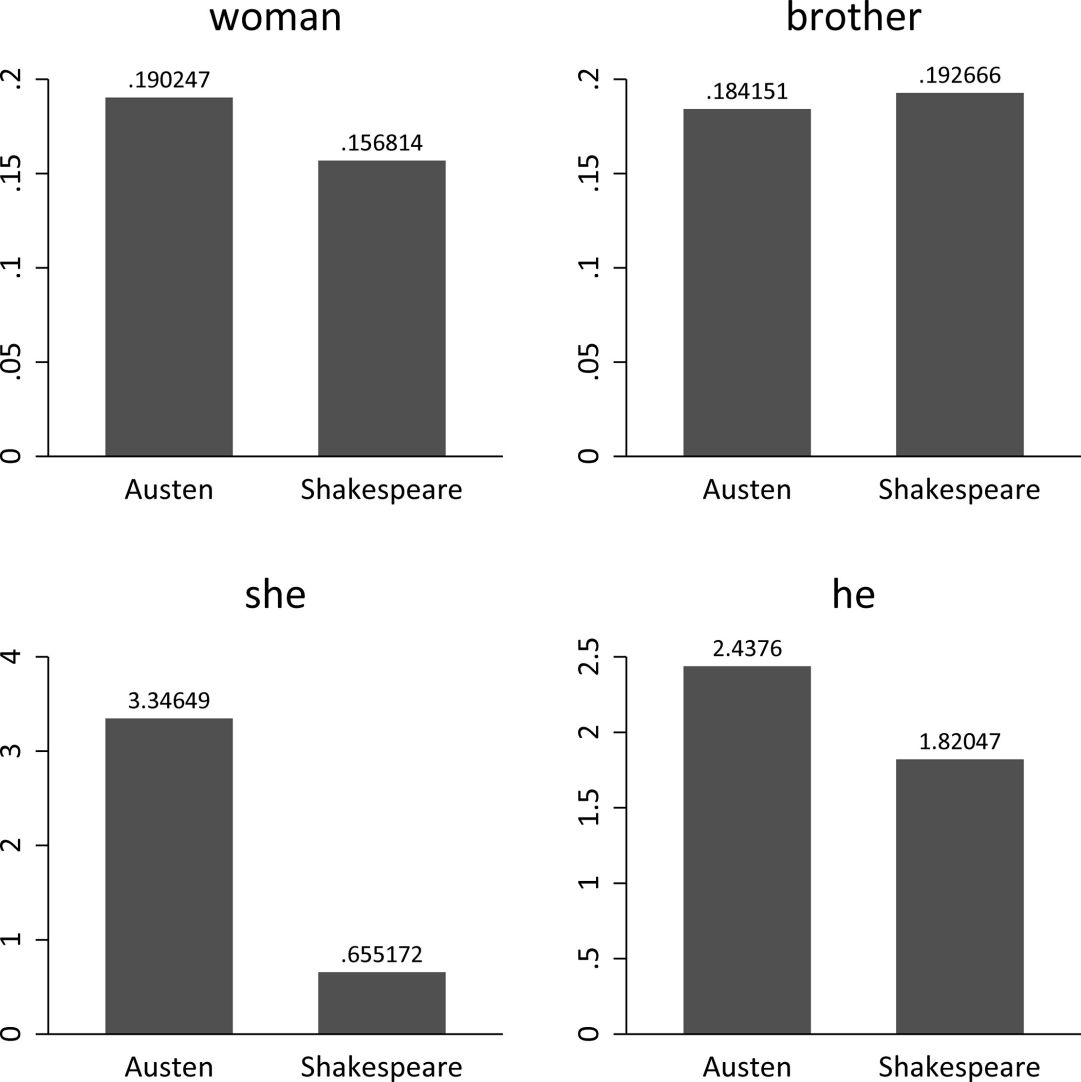
Frequencies per page for four selected words in the Austen and in the Shakespeare corpus.

Thus, with *N* = 250, the mean of *f*(*w*) quantifies the average word frequency per page. Again, the advantage is that we do not have to settle for just two data points, but can use the available information to conduct a *t-*test. Fig 2 plots the frequencies and Table 1 summarizes the corresponding *p-*values.

**Table 1:** *p-*values (one-sided) based on *t*-tests on the equality of means for the four selected words in the Austen and in the Shakespeare corpus.

Word	*p*-value
Brother	.270
He	.000
She	.000
Woman	.004

As above, it is hard to see a coherent interpretation of the computed significance levels, since parametric statistical inferences require random sampling as written above, something that is clearly not the case here. Or put differently, how can we regard our corpus data from Austen and Shakespeare as a random sample (or a sample at all) of any population?

Nevertheless, this is another example of a situation where a researcher would like to attach some kind of statistic to the results in order to quantify whether it is *substantial* or *accidental*. This is especially true in situations where several quantities are compared with each other (cf. Section 6). This leads me to the next section.

## 4 The permutation test

As argued above, classical null hypothesis significance tests are based on a number of assumptions that are (i) in almost all cases violated in corpus linguistics and (ii) in some cases, like in the examples presented above, the purpose of a test is not to extend the quantities found in one corpus to the language it seeks to represent, but to determine whether a result is pronounced enough to warrant the conclusion that it is *substantial* and not *due to chance*. Regarding (ii), I demonstrate in this section that *permutation tests* can be used in such situations.

Compared to classical significance null hypothesis tests, permutation tests are a class of non-parametric tests [62,64]. Or put differently, while classical tests assume that under the null hypothesis the investigated data follow a specific distribution (e.g. independent, normally and identically distributed *errors*), permutation tests use the data itself to compute the distribution of the test statistic under the null hypothesis that is spelled out explicitly. It is important to point out that permutation testing is not meant as a substitute for the construction of plausible statistical models for the observed data, but offers a non-parametric way to directly compute the reference distribution of any test statistic from which the *p*-values can be obtained. As such, permutation tests can be understood as a procedure for accepting or rejecting hypotheses based on statistical models with various degrees of complexity. To outline the procedure for corpus linguistic data, a simple but important example is presented in this paper, the comparison of word frequencies. For an application with linguistic data that tests hypotheses that are based on linear effects models, see [65]. A standard comprehensive textbook reference is [66].

The development of permutation tests can be traced back to Fisher [67] and Pitman [68]. The following interesting quote from Fisher [69], (one of) the founding father(s) of modern statistics (brought to my attention by reading [62]) illustrates the most basic idea of permutation testing:

“Let us suppose, for example, that we have measurements of the stature of a hundred Englishmen and a hundred Frenchmen. It may be that the first group are, on the average, an inch taller than the second, although the two sets of heights will overlap widely. […] The simplest way of understanding quite rigorously, yet without mathematics, what the calculations of the test of significance amount to, is to *consider what would happen if our two hundred actual measurements were written on cards*, *shuffled without regard to nationality*, *and divided at random into two new groups of a hundred each*. *This division could be done in an enormous number of ways*, *but though the number is enormous it is a finite and a calculable number*. We may suppose that for each of these ways the difference between the two average statures is calculated. Sometimes it will be less than an inch, sometimes greater. If it is very seldom greater than an inch, in only one hundredth, for example, of the ways in which the sub-division can possibly be made, the statistician will have been right in saying that the samples differed significantly. *For if*, *in fact*, *the two populations were homogeneous*, *there would be nothing to distinguish the particular subdivision in which the Frenchmen are separated from the Englishmen from among the aggregate of the other possible separations which might have been made*. Actually, the statistician does not carry out this very simple and very tedious process, but his conclusions have no justification beyond the fact that they agree with those which could have been arrived at by this elementary method.” (my emphasis)

Interestingly, this can be adapted directly to the data representation outlined in the last section: the quantities of interest are word frequencies that are compared between two ‘books’, i.e. corpora. On each page, each token of a word type of interest is highlighted. To determine the significance of a difference, the researcher can simply shuffle all *pages without regard to* authorship, and divide the pages *into two new* ‘books’, with the group size being equal to the original distribution. She could then calculate the difference between the means. This procedure could be repeated very often, say 100,000 times, resulting in 100,000 differences. At the time of writing, Fisher was, of course, right that *carrying out this* procedure would be a *very tedious process*. Luckily, nowadays the researcher can rely on fast and inexpensive computers in order to take care of the *tedious* part. After the computer produced a certain amount of random (‘Monte Carlo’) permutations, the researcher could then count the number of times when the difference in means for the randomly permuted arrangement *is at least as big as* the difference between the means computed on the original data. If we denote that number as *d*, then the *p*-value is computed as *d*/100,000. If the researcher opts for a significance level of *p* < .01, then a difference would only be labeled as *significant* or *substantial* if *d* < 1,000.

A simple hypothetical example illustrates the basic idea of permutation tests: assume that we want to compare the lexical richness of two short speeches of two different authors (*Author 1* and *Author 2*), each with a length of 750 words. If we choose a segment length of *N =* 250, we can calculate the number of different word types for each of the three resulting segments or pages. For *Author 1*, we receive: 70, 74, 72 and for *Author 2*: 67, 69, 71. In order to find out if the difference in means of (70+74+72)/3 –(67+69+71)/3 = 3.00 is *substantial* or *accidental*, we can conduct a permutation test that evaluates *what would happen if the pages were shuffled without regard to authorship*, *and divided at random into two new groups of three each*. In a certain sense, this random re-arrangement simulates the null hypothesis, i.e. that the lexical richness is not different between *Author 1* and *Author 2*. If this is the case, then the authorship label (*Author 1* or *Author 2*) becomes arbitrary and thus can be randomly re-arranged, i.e. permuted. A permutation test with 1,000 repetitions returns the following result: in *d* = 100 cases, the difference between the two means is at least as big as the observed difference in means of 3. Thus, the *p*-value is 100/1000 = .10. This value implies that in 10% of all permutations, we find a difference that is as least as big as the difference that was actually observed. Hence, the observed difference can be considered to be *accidental*. This is the general interpretation of a permutation-based *p*-value: it measures the probability of finding a test statistic that is as least as high as the one that was actually observed, given that the null hypothesis is true, i.e. there is no difference between the compared groups.

Note that we could also have calculated the *t*-statistic for each permutation (cf. Eq 3). However, this is equivalent to using the difference in means as the test statistic here, since the other quantities of interest (cf. Eq 3 and Eq 4) remain constant across permutations.

Since the number of cases in this example is very small, we can calculate an exact permutation *p*-value. In Table 2, all $\left(\begin{array}{c} 6\\ 3 \end{array}\right)=20$ possible unique permutations are listed. In each case, the test statistic of interest *T*, i.e. the obtained mean difference is calculated. The rows in Table 2 are then ranked in descending order based on the *T*-values. The rank of the observed difference determines its significance: Two of the 20 permutations display a difference of at least 3.00, so the rank of the observed difference (highlighted in bold face) is 2 and the exact *p*-value can be calculated as 2/20 = 0.10. However, the number of possible unique permutations for larger data sizes quickly becomes enormous. In these cases, *p-*values can be estimated on the basis of Monte Carlo simulations by randomly permuting the values of the variable of interest a large number of times as described above.

**Table 2:** Example of all possible unique permutations for the hypothetical example.

Author 1			Mean	Author 2			Mean	*T*	Rank
71	72	74	72.33	67	69	70	68.67	3.67	1
**70**	**74**	**72**	**72.00**	**67**	**69**	**71**	**69.00**	**3.00**	**2**
70	71	74	71.67	67	69	72	69.33	2.33	3
69	72	74	71.67	67	70	71	69.33	2.33	4
69	71	74	71.33	67	70	72	69.67	1.67	5
70	71	72	71.00	67	69	74	70.00	1.00	6
69	70	74	71.00	67	71	72	70.00	1.00	7
67	72	74	71.00	69	70	71	70.00	1.00	8
69	71	72	70.67	67	70	74	70.33	0.33	9
67	71	74	70.67	69	70	72	70.33	0.33	10
69	70	72	70.33	67	71	74	70.67	-0.33	11
67	70	74	70.33	69	71	72	70.67	-0.33	12
69	70	71	70.00	67	72	74	71.00	-1.00	13
67	71	72	70.00	69	70	74	71.00	-1.00	14
67	69	74	70.00	70	71	72	71.00	-1.00	15
67	70	72	69.67	69	71	74	71.33	-1.67	16
67	70	71	69.33	69	72	74	71.67	-2.33	17
67	69	72	69.33	70	71	74	71.67	-2.33	18
67	69	71	69.00	70	72	74	72.00	-3.00	19
67	69	70	68.67	71	72	74	72.33	-3.67	20

For the examples presented in Section 3, permutation tests can help to answer the question whether a result is pronounced enough to warrant the conclusion that it is *substantial* and not *due to chance*. In the following section, the test results are presented.

## 5 Results

Freedman and Lane [62] give a general outline of the procedure described in the preceding section:

- Data are obtained in a non-parametric situation. The researcher wants to answer the question whether one particular attribute of the data can “be dismissed as an artifact, or does it require a more substantial explanation?” In the example of comparing Austen with Shakespeare, the attributes of interest are the lexical richness or certain word frequencies.
- A test statistic is computed. The “larger the value of the test statistic”, “the more pronounced” the attribute of interest. In the examples, the test statistics are the mean differences in lexical richness and in relative word frequencies.
- A “new class of data sets is introduced”: to calculate the distribution under the null hypothesis, i.e. data sets where the attribute of interest is only *accidental*, but that are “otherwise comparable to the original data”. In the examples, the new data sets are derived by randomly sorting the *pages*. For each derivation, the first 7,793 pages are labeled as belonging to the ‘Austen’ corpus, while the remaining 9,137 pages are labeled as ‘Shakespeare’.
- For each data set derived in (3), the test statistic from (2) is computed.
- “The relative position of the test statistic for the original data set compared to all the values calculated in (4) is determined.”

For the comparison of the Austen and the Shakespeare corpus regarding the lexical richness (cf. Fig 1), we find that the difference of 0.567–0.563 = 0.004 is pronounced enough to label this result as *substantial*: only 3 of 100,000 random permutations of the *TTR*-per-page values display a difference that is at least as big as the one actually observed. For the mock data set with a difference between the means of 0.5655–0.5643 = 0.0012, we find that 13,528 of 100,000 random permutations have a least a difference of 0.0012. Thus, as *p* = .14 and the significance level is 1%, the researcher would conclude that this difference is *accidental*.

Regarding the differences in word frequencies, Table 3 presents the results of separate permutation tests with 100,000 repetitions.

**Table 3:** Results of separate Monte Carlo permutation tests with 100,000 repetitions on the equality of means for the four selected words in the Austen and in the Shakespeare corpus.

Word	*d*	*p*-value
Brother	27004	.270
He	0	.0000
She	0	.0000
Woman	426	.004

Thus, we can say that all differences are substantial or significant at *p* < .01, except for the difference for ‘brother’ (*p* = .270). (*NB*.: it is worth pointing out that since multiple tests are being conducted here, adjusting the significance level, e.g. Bonferroni normalization, makes sense. For instance, four tests were performed here, each at the *p* < .01 level, then the Bonferroni adjustment would lower this level to 0.01/4 = .0025. In this case we would conclude that the difference for ‘woman’ is *accidental*.)

If we compare the results of the *t*-tests with the results of the permutation tests, we see a remarkable similarity. For example, the permutation test for the simulated *TTR* values returned a *p*-value of .133 compared with a *p-*value of .135 based on a standard *t*-test presented above. Or, for the word ‘she’, the *t*-test based *p*-value of .00411 closely matches the permutation based *p*-value of .00426.

As it turns out, this is more than just a coincidence as shown in the next section.

## 6 *t*-tests as an approximation and several remarks

As written above, with the availability of both affordable and fast computers, today permutation tests offer a real alternative to standard frequentist approaches. However, it is important to point out that the statistic of interest has to be computed for each permutation in addition to actually permuting the data. Therefore, in situations where multiple tests are being conducted, computing time can still be a major issue. In those situations, a theorem by Wald and Wolfowitz [70] can be useful as an approximation: amongst others, Freedman and Lane [62,71] analytically prove that the *p-*value from a *t*-test locates (as in Table 2) the observed data among the *n*! permuted data sets (y*,*z*). Here, the relationship between the *t*-value and the correlation coefficient *r* (cf. Eq 3) is illustrative: if *y* is unrelated to *z*, then we would expect *r* to be 0 and any deviation from 0 to be purely accidental.

Again, the idea is that if there is *nothing to distinguish* between the two groups, then the group label does not matter, so the derived data sets that are generated by randomly permuting the data should be *indistinguishable* from the original data. Or put differently, the *p*-value of a *t*-test can be interpreted as approximately reporting the fraction of permutations for which $r_{y^{*}z}$ is greater than the observed *r*_*yz*_.

This ‘permutation interpretation’ of standard *t-*test-based *p*-values is appealing since it does not make any “assumptions about the mechanism for generating the data” [71]. To see that this approximation is also useful in the context of comparing word frequencies, I compared the results of a *t-*test with permutation tests each with 1,000 repetitions for all words in the Austen and Shakespeare corpus with an absolute frequency of at least two in the concatenation of the two corpora. For the resulting 16,588 words, Table 4 summarizes the agreement between both test methods. The rate of agreement is very good, in 96.60% of all cases, both tests came to the same conclusion. It can be expected that this rate will be even higher when the number of replications is increased. For example, the *t*-test based *p-*value for the word ‘woman’ is .0041 (cf. Table 1).

**Table 4:** Agreement between the *t*-test and the permutation test. ‘No’ and ‘Yes’ indicate that the corresponding *p-*value is greater than or equal to (= ‘No’) or below (= ‘Yes’) the significance level of 1%.

Significant?		Permutation test		
		No	Yes	Total
***t*-test**	No	10,460 95.45%	65 1.15%	10,525 63.45%
	Yes	499 4.55%	5,564 98.85%	6,063 36.55%
	Total	10,959 100.00%	5,629 100.00%	16,588 100.00%

With 1,000 replications, the corresponding permutation-based *p-*value is .0070. However, with 100,000 replications (cf. Table 3), the permutation-based value of .0042 is already much closer to the *t*-based *p*-value. The same can be said for the word ‘brother’: the *t*-based *p*-value of .2635 is closer to the permutation-based *p*-value with 100,000 repetitions (.2700) compared to the *p*-value with 1,000 repetitions (.2520).

Thus, in situations where a permutation-based approach is computationally too expensive, a *permutation* interpretation of the *p*-values from *t*-tests can be used.

Before concluding, five points are worth mentioning:

- Word frequencies in corpora and texts often tend to be underdispersed [63,72], i.e. the tokens of a particular word type are not evenly distributed across the whole corpus. A simple hypothetical example demonstrates that a data representation like the one outlined in Section 3 in conjunction with a permutation test can also be useful in such a situation: assume that we want to compare the word frequency of a particular word in two short speeches of two different authors (*Author 1* and *Author 2*), each with a length of 1,250 words. If we choose a segment length of *N =* 250, we can calculate the word frequencies for each of the five resulting pages as described above. For *Author 1*, we receive: 2, 2, 2, 2, 2 and for *Author 2*: 1, 1, 1, 1, 1. So in this case, the word frequency is well dispersed across the segments. To find out if the difference in mean frequency of (2+2+2+2+2)/5 - (1+1+1+1+1)/5 = 1 is *substantial*, we can conduct a permutation with 1,000 repetitions. A *p-*value of .005 warrants the conclusion of a *substantial* difference. Compare this to the following distribution: for *Author 1*, we receive: 1, 1, 1, 1, 6 and for *Author 2*: 1, 1, 1, 1, 1. Here, the word frequency for *Author 1* is distributed very unevenly. However, since (1+1+1+1+6)/5 - (1+1+1+1+1)/5 = 1, the difference in mean frequency is the same as in the first scenario. In this case, it is less clear whether we would call this difference *substantial* as the word frequencies are very similar, except for one outlier. Correspondingly, a permutation with 1,000 repetitions returns a *p*-value of .515. By only looking at the mean frequencies in isolation, this difference in dispersion might have been overlooked.
- From a statistical point of view, the segment length of *N* words (cf. Section 3) is a *free* parameter. As argued above, *N* = 250 is a natural choice since the segment length can then be understood as *one standard page*. Nevertheless, it stands to reason that the conclusions presented in this paper remain comparable with a different segment length. To this end, I re-ran all analyses with three further segment lengths, *N =* 100, *N* = 1,000 and *N* = 10,000. Regarding the different attributes of interest, Table 5 shows that the conclusions reached on the basis of different segment lengths are qualitatively very similar except for the word frequency comparison for ‘woman’, where a *substantial* effect, i.e. *p* < .01 can only be deduced with a segment length of *N* = 100 and *N* = 250. This demonstrates that it can make sense to calculate results based on different segment lengths, as already suggested by Johnson [60].
- Another parameter that the researcher has to decide upon is the number of permutations. In general, it is possible to compute confidence intervals for the *p*-value based on the binomial distribution [73]. If the confidence interval indicates that it is uncertain that a *p*-value falls below a chosen significance level, it makes sense to increase the number of permutations, because the *p*-value will converge to the true permutation *p*-value (cf. Table 2) with the number of permutations. For instance, imagine a situation where a permutation test with 1,000 repetitions returns *p* = .009. In this case, the exact 95% binomial confidence interval is [.0041; .0170]. In this case, it is somewhat unclear if the null hypothesis can be rejected at *p* < .01. If we increase the number of permutations to 100,000 the interval for *p* = .009 will be [.0084; .0096] and thus warrants the rejection of the null hypothesis at *p* < .01. There are interesting approaches to automatically determining the required number of permutations via stopping rules (cf. e.g., [74]).
- Since word frequency distributions can be characterized by a large number of rare events [58], there will be many cases where the number of unique permutations will be sharply reduced, because the respective words occur very rarely. For example, the word ‘printed’ occurs twice in total, both in the Austen corpus and on two separate pages. In this case, we can calculate the exact permutation value that is given as the probability that none of those two pages on which ‘printed’ occurs belong to the Shakespeare corpus, by using combinatorics:
  p=∏i=1knn+1−in+1−i=(nnk)(nk)=nn!{k!*(nn−k)!}n!{k!*(n−k)!} (8)
  where *n* denotes the number of available pages in the concatenation of both corpora, *n*_*n*_ denotes the subset of available pages *without* an occurrence of the word of interest and *k* denotes the number of available pages in the Shakespeare corpus. Plugging-in the corresponding numbers yields an exact *p-*value of .2118. A Monte Carlo permutation test with 100,000 repetitions comes very close to this value (*p* = .2122), while a *t*-test strongly underestimates the exact probability (*p* = .0629).
- Throughout this paper, *t*-tests were conducted with the implicit assumption of equal variances. Therefore, I re-ran all *t-*tests with the unequal variance option [75]. All results remain qualitatively the same.

**Table 5:** Results for permutation test with different segment lengths.

Attribute	*p*-value with *N* = 100	*p*-value with *N* = 250	*p*-value with *N* = 1,000	*p*-value with *N* = 10,000
brother	.24437	.27004	.31753	.36108
he	0	0	0	0
she	0	0	0	0
woman	.00178	.00426	.01196	.04454
lexical richness	0	0	0	0
Agreement rate with *t-*test for 16,588 words	.96594	.96600	.95599	.95720

## 7 Conclusion

Permutation tests have seen a rise in popularity in recent years. On the one hand, this certainly has to do with the aforementioned fact of the current availability of cheap and fast computing. On the other hand, permutation tests offer a real alternative to standard parametric null hypothesis significance tests, the assumptions of which are in reality often very hard to meet. Permutation tests only make minimal assumptions, i.e. exchangeability under the null hypothesis. Informally speaking, this means that randomizing data points ensures that the data remains just as likely as the originally observed data. This assumption can be critical when there is dependency among observations, for example when the data is temporally ordered in the case of a diachronic study. In such a situation, it is important to be careful when selecting what and how to permute [76].

For corpus linguistic and textual data, I hope that I was able to demonstrate that permutation tests offer interesting opportunities. For more complex test scenarios as the ones presented in this paper, various approaches have been produced and tested in different fields [64,77–79]. For instance, Freedman and Lane [71] and Still and White [80] suggest permuting residuals in a model where potential confounding variables are regressed-out in order to test the significance of a variable of interest in a multiple variable scenario. For an application, cf. [81].

In situations where multiple tests are involved, permutation tests might still be too expensive to conduct. In those cases, the theorem of Wald and Wolfowitz [70] and the extension of Freedman and Lane [71] can be used to interpret *p-*values that are based on a standard *t*-test in a non-stochastic ‘permutation’ fashion. The *p*-value can then be understood as a descriptive statistic: “a small reported significance level indicates an unusual data set” [71].

I believe that one of the less obvious advantages of permutation tests is a methodological one: it forces the researcher to explicitly spell out the null hypothesis in parallel to the long Fisher quotation above: if a variable of interest (e.g. the authorship label) does *nothing to distinguish*, it can be permuted. This then leads to a *randomized* experiment where the computer takes care of the *tedious* part of deriving a spectrum of other data sets from the observed data [71]: if there is no difference in frequency for a particular word type between two books or corpora, the books with permuted pages should, more or less, look like the original two books regarding this attribute. If this is not the case, then this might indicate a difference that is *substantial*.

So, the next time I am asked what to do if the aim of a study is to judge the relevance of a result that is based on corpus linguistic data, I will reply: *try permutation tests*, both as a statistical method to answer the question and, on a more general level, as a method of scientific reasoning in order to better understand both the answer and the question.

## Supporting information

S1 Code Stata (14) code. Code required to reproduce all results presented in the paper.
(ZIP)
